# The prevalence of early contained vascular injury of spleen

**DOI:** 10.1038/s41598-024-58626-2

**Published:** 2024-04-04

**Authors:** Seppo K. Koskinen, Z. Alagic, A. Enocson, A. Kistner

**Affiliations:** 1 Division for Radiology, Department of Clinical Science, Intervention, and Technology, Karolinska Institutet, 171 76 Stockholm, Sweden; 2 Department of Diagnostic Radiology, Karolinska University Hospital, 171 76 Stockholm, Sweden; 3 Department of Molecular Medicine and Surgery, Karolinska Institutet, 171 76 Stockholm, Sweden; 4 Department of Trauma, Acute Surgery and Orthopaedics, Karolinska University Hospital, 171 77 Stockholm, Sweden; 5 Medical Radiation Physics and Nuclear Medicine, Karolinska University Hospital, Stockholm, Sweden

**Keywords:** Traumatic abdominal injury, CT scans, Spleen, Pseudoaneurysm, Arteriovenous fistula, Contained vascular injury, Epidemiology, Medical imaging, Health care, Medical research

## Abstract

Contained vascular injuries (CVI) of spleen include pseudoaneurysms (PSA) and arterio-venous fistulae (AV-fistulae), and their reported prevalence varies. Our purpose was to assess the prevalence of early splenic CVI seen on admission CT in patients with splenic trauma admitted to a single level 1 trauma center in 2013–2021, and its detection in different CT protocols. A retrospective, single-center longitudinal cohort study. Nine-year data (2013–2021) of all patients with suspected or manifest abdominal trauma were retrieved. All patients, > 15 years with an ICD code for splenic trauma (S36.0XX) were included. CT and angiographic examinations were identified. Reports and images were reviewed. Splenic CVI CT criterion was a focal collection of vascular contrast that decreases in attenuation with delayed imaging. Number of CVIs and treatment was based on medical records and/or available angioembolization data. Of 2805 patients with abdominal trauma, 313 patients (313/2805; 11.2%) fulfilled the study entry criteria. 256 patients (256/313; 81.8%) had a CT examination. Sixteen patients had splenectomy before CT, and the final study group included 240 patients (240/313; 76.7%). Median New Injury Severity Score (NISS) was 27 and 87.5% of patients had NISS > 15. Splenic CVI was found in 20 patients, which yields a prevalence of 8.3% (20/240; 95% CI 5.2–12.6%). In those cases with both late arterial and venous phase images available, CVI was seen in 14.5% of cases (18/124, 95% CI 8.6–22.0%). None of the patients with CVI died within 30 days of the injury. The prevalence of early splenic CVI in patients with a splenic trauma was 8.3–14.5% (95% CI 5.2–22.0%). Our data suggests that both arterial and venous phase are needed for CT diagnosis. The 30-day outcome in terms of mortality was good.

## Introduction

Blunt splenic injury occurs in 2% of all trauma admissions, and splenic rupture is the most common cause of major abdominal injury in the vast majority of cases^1,2^. It is the most vascular organ in the abdomen, and the bleeding is usually arterial causing a major hemoperitoneum and severe blood loss leading to hemodynamic instability and even death^3^. Therefore, the detection of active splenic hemorrhage and contained vascular injuries is crucial for identifying the need for either subsequent direct intervention (surgery or transcatheter embolization), or if conservative nonsurgical treatment can be applied^4–8^. Because of the spleen’s immunological function, salvage rather than removal is the desirable treatment option^9^.

CT with intravenous contrast is currently the imaging method of choice in major blunt trauma^10^. It can be used to classify abdominal organ trauma according to the AAST (American Association for the Surgery of Trauma) Injury Grading Scale. The most recent (2018) revision of this^11^ includes vascular injuries such as traumatic splenic pseudoaneurysms (PSA) and arteriovenous (AV) fistulae. These appear on CT as a focal collection of vascular contrast that decreases in attenuation with delayed imaging. Traumatic PSA and an AV-fistula may be impossible to differentiate on CT, and these findings are best described together as those of contained vascular injury (CVI)^6,12,13^.

To diagnose CVI, special attention has to be addressed to the way contrast agent is administered. The current recommendation is to image the spleen in both arterial and venous phase^13,14^. If only venous phase is used, these injuries may go unrecognized. Similarly, if the so-called “split bolus” technique is used, it may be impossible to differentiate active bleeding from contained injury^15^.

In the past, the true prevalence of early splenic CVI has been difficult to establish, but the improvements in CT technology paired with trends toward more comprehensive imaging workups of trauma has led to an increase in CVI detection^16^. The reported prevalence of CVI in acute phase varies, but it has been reported to occur in up to 20% in patients with splenic trauma^14,16^. Therefore, the purpose of this study was to find out the prevalence of early splenic CVI seen on admission CT in patients with splenic trauma admitted to a single level 1 trauma center in 2013–2021, and its detection in different CT protocols.

## Methods

Approval from the local ethics committee was obtained for this retrospective, longitudinal cohort study (Ethical application number *2019-05643, 2022-02753-02*) and due to its retrospective design, informed consent was waived. Nine-year data (2013–2021) of all consecutive patients with suspected or manifest abdominal trauma were retrieved from the local trauma register at the Karolinska University Hospital, a level 1 trauma center with a total catchment area of approximately 2.5 million people. The individual patient data is coded in the registry by trained nurses with several years of experience. The data is registered according to the The Utstein Trauma Template for Uniform Reporting of Data following Major Trauma^17^. The extraction of data from the register is acknowledged and supervised by the local research council. To ensure that all patients with abdominal trauma could be identified, the initial dataset from the register included all trauma patients admitted with either an AIS-code for body region 5 or an ICD code S3X.XX. All patients, 15 years and older with an ICD code for splenic trauma (S36.0XX) were included. Both blunt and penetrating trauma were included. The following parameters were extracted from the trauma register and/or electronic medical records: age, gender, Injury Severity Score (ISS), New Injury Severity Score (NISS), 30-day mortality, injury mechanism^17^, surgical and/or radiological interventions (angioembolization), and length of hospital stay (LOS).

The CT images were retrieved from the local picture archiving and communication systems (the Radiological Information System (RIS)/Picture Archiving and Communication System (PACS); SECTRA AB, Linköping, Sweden), and the CT protocol was recorded. Also, patients with CT scans from other hospitals were included.

CT and angiographic examinations from all eligible patients were identified and the images as well as imaging and clinical reports were reviewed by a senior radiologist (SKK) with 30 years of experience. All CT scans had previously been interpreted by at least one radiology resident and a senior radiologist.

The splenic traumas seen on CT were graded according to AAST Injury Grading Scale 1994 and 2018 revisions. For splenic CVI identified in CT, we used the criterion described earlier, i.e., a focal collection of vascular contrast that decreases in attenuation with delayed imaging^13^. If a CVI was suspected, a second radiologist (ZA) reviewed the cases, and any discrepancy was solved by consensus.

The reference standard for CVI detection was angioembolization data, and in instances where this was unavailable, the reference standard comprised follow-up CT and information from medical records.

### CT technique

At our hospital, the multitrauma imaging was conducted on a 256-slice multi-detector CT (MDCT) (Revolution CT, GE Healthcare, Milwaukee, Wisconsin, USA) and a 64-slice MDCT scanner (LightSpeed VCT, GE Healthcare, Milwaukee, Wisconsin, USA). During 2015 the Revolution CT scanner (RevCT) was installed and replaced the LightSpeed VCT scanner (VCT) in our trauma department. Concomitantly our single-phase (venous) standard trauma CT protocol was modified to a multi-phase protocol by including a whole-body CT in arterial phase for high-energy trauma patients. The imaging parameters are presented in Tables 1 and 2.

**Table 1:** Scanning parameters for LightSpeed VCT.

Scanning parameter	CT thorax and abdomen in venous phase
Tube current (mA)	225 (300 for obese), fixed
Tube voltage (kVp)	120 (120 for obese), fixed
Rotation time (sec.)	0.5
Helical pitch	0.992
Detector coverage (mm)	80
Reconstruction algorithm	ASiR-V™ 50%
Convolution kernel	Standard
Slice thickness (mm)	0.625
Delay (sec)	65
Iodinated contrast agent	Ioversol/Iohexol 350 mg/ml
Contrast agent dose	500 mgI/kg*
Injection time (sec)	30

*The contrast was dosed at 500 mgI/kg with a maximum dosage weight of 80 kg for women and 100 kg for men.

**Table 2:** Scanning parameters for RevCT.

Scanning parameter (high-energy trauma)	Whole-body CT in arterial phase	CT abdomen in venous phase
Tube current (mA)	150 (300 for obese), fixed	120 (255 for obese), fixed
Tube voltage (kVp)	100 (120 for obese), fixed	120 (120 for obese), fixed
Rotation time (sec.)	0.5	0.5
Helical pitch	0.992	0.992
Detector coverage (mm)	80	80
Reconstruction algorithm	ASiR-V™ 50%	ASiR-V™ 50%
Convolution kernel	Standard	Standard
Slice thickness (mm)	0.625	0.625
Delay (sec)	SmartPrep™	45 (after end of arterial phase)
Iodinated contrast agent	Ioversol/Iohexol 350 mg/ml	Ioversol/Iohexol 350 mg/ml
Contrast agent dose	500 mgI/kg*	500 mgI/kg*
Injection time (sec)	20	20

Scanning parameter (low-energy trauma)	CT thorax and abdomen in venous phase	
Tube current (mA)	225 (300 for obese), fixed	
Tube voltage (kVp)	120 (120 for obese), fixed	
Rotation time (sec.)	0.5	
Helical pitch	0.992	
Detector coverage (mm)	80	
Reconstruction algorithm	ASiR-V™ 50%	
Convolution kernel	Standard	
Slice thickness (mm)	0.625	
Delay (sec)	65	
Iodinated contrast agent	Ioversol/Iohexol 350 mg/ml	
Contrast agent dose	500 mgI/kg*	
Injection time (sec)	30	

*The contrast was dosed at 500 mgI/kg with a maximum dosage weight of 80 kg for women and 100 kg for men.

### Statistics

Mann–Whitney U test for independent samples and chi-square test were used to calculate the difference between patients with and without splenic CVI. Confidence intervals were calculated using binomial exact test. Statistical analyses were done using a commercial software package SAS/STAT v.9.4 (SAS Institute Inc., Cary, NC, USA).

### Ethical approval

Approval from the local ethics committee was obtained for this retrospective, longitudinal cohort study (Swedish Ethical Review Authority, Ethical application number 2019-05643, 2022-02753-02).

### Consent to participate

Informed consent was waived by Swedish Ethical Review Authority due to the retrospective design. All methods were carried out in accordance with relevant guidelines and regulations. All experimental protocols were approved by Karolinska University Hospital’s Internal Review Board.

## Results

During the nine-year study period, a total of 2805 patients were admitted to the trauma center following abdominal trauma.

A total of 313 patients (313/2805; 11.2%) had splenic trauma, and 256 (256/313; 81.8%) had undergone a CT on admission. Of the 57 cases with no CT, 13 were dead on admission, and in the remaining 44 cases, no CT or angiographic examinations on admission could be found or retrieved. Furthermore, sixteen patients had had a splenectomy before CT, so the final study group included 240 patients (240/313; 76.7%) (Table 3). The main injury mechanism was motor vehicle collision (MVC) in 90 cases (37.5%), followed by fall from height in 62 cases (25.8%) and blunt trauma to the body in 22 cases (9.2%). The yearly prevalence is presented in Table 4. The CT protocol included both arterial and venous phase in 124 (124/240; 51.7%) cases and a venous phase only in 107 (107/240; 44.6%) cases. The remaining 9 (9/240; 3.7%) presented with different variations of CT protocols (Table 5).

**Table 3:** Patient characteristics.

Characteristics	
No. of patients (n)	240
Age (years)	40.1 (15–92)
Sex	
Male	184 (76.7%)
Female	56 (23.3%)
ISS	24 (4–66)
ISS > 15 (n)	195 (81.3%)
NISS	27 (4–75)
NISS > 15 (n)	210 (87.5%)
LOS days	12.3 (1–86)
Survived to discharge (reciprocal of 30-day mortality)*	227 (94.6%)

Values are given in mean (age, LOS) or median (ISS, NISS). Range in parentheses.

PA, splenic pseudoaneurysm; ISS, Injury Severity Score; NISS, New Injury Severity Score; LOS, hospital length of stay.

*In one case this information was missing.

**Table 4:** Number of abdominal injuries and splenic injuries with CT available and no previous splenectomy, and the proportion of these splenic injuries (ICD code S36.0XX each year).

Year	Abdominal injuries	Splenic injuries CT available	%
2013	275	31	11.3
2014	280	25	8.9
2015	252	18	7.1
2016	268	23	8.6
2017	341	21	6.2
2018	327	13	4.0
2019	349	40	11.5
2020	368	38	10.3
2021	345	31	9.0
Total	2805	240	8.6

**Table 5:** Whole body trauma CT protocols.

CT protocol/phase	N	(%)
Arterial and venous	119	49.6
Venous only	107	44.6
Arterial and delayed images	1	0.4
Arterial and venous and repeated venous phase	5	2.1
No contrast	1	0.4
Single bolus	6	2.5
Arterial and delayed images and later a venous phase	1	0.4
Total	240	100

The grading of splenic injuries according to AAST OIS 1994 and 2018 systems are presented in Tables 6 and 7. The most common injury was Grade III using the 1994 revision, whereas Grade III and IV equaled using the 2018 revision.

**Table 6:** Splenic injury grade according to AAST OIS 1994 revision.

Grade (1994)	N (%)	Reference data^a^ N (%)	Reference data^b^ N (%)
I	27 (11.25)	37 (14.07)	38 (10.89)
II	56 (23.33)	67 (25.48)	65 (18.62)
III	96 (40.00)	107 (40.68)	170 (48.71)
IV	41 (17.08)	45 (17.11)	47 (13.47)
V	20 (8.33)	7 (2.66)	29 (8.31)
Total	240	263	349

^a^ and ^b^Data are from reference^19^ and^20^, respectively.

**Table 7:** Splenic injury grade according to AAST OIS 2018 revision.

Grade (2018)	N (%)	Reference data^a^ N (%)
I	27 (11.25)	38 (10.89)
II	52 (21.67)	63 (18.05)
III	64 (26.67)	137 (39.26)
IV	62 (25.83)	58 (16.62)
V	35 (14.58)	53 (15.19)
Total	240	349

^a^Data are from reference^20^.

In 66 cases, an angioembolization was performed with either coils or Amplatz plugs (G II 1; G III 10; G IV 39 and G V 16 cases. Grading according to AAST OIS 2018 revision). In one case a collagen fleece (TachoSil) was applied.

### Contained vascular injuries

**Figure 1:**
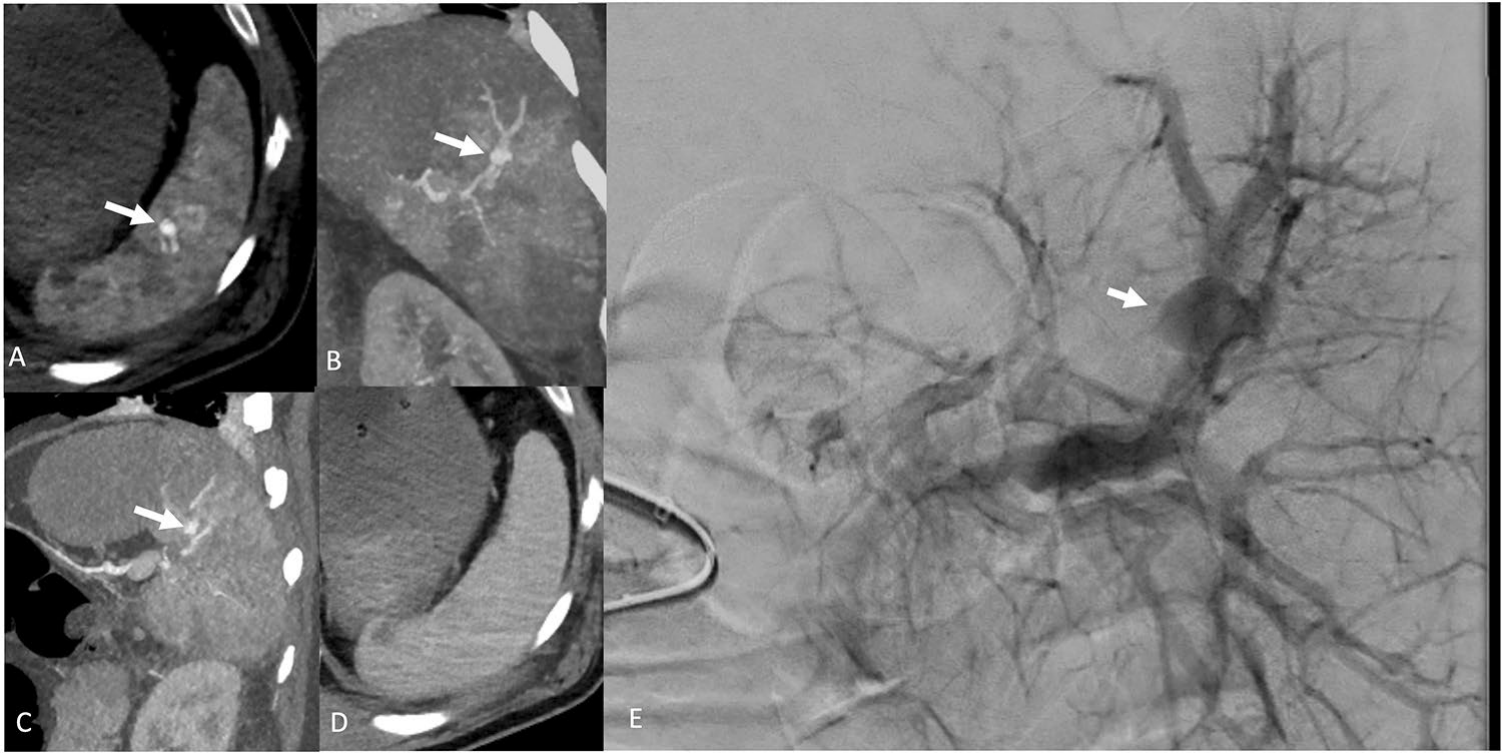
A 55-year-old male involved in a motor vehicle accident with multiple rib fractures and G III kidney injury (not shown), ISS 26. Contrast-enhanced CT scans (**A**–**D**). Axial, coronal, and sagittal images in (**A**–**C**), arterial and (**D**) portal venous phases. Images in arterial phase show indistinct area of hyperattenuating foci (arrows). This area washes out at portal venous phase. Patient underwent angiography (**E**), and contained vascular injury (arrow) was subsequently embolized (not shown).

**Figure 2:**
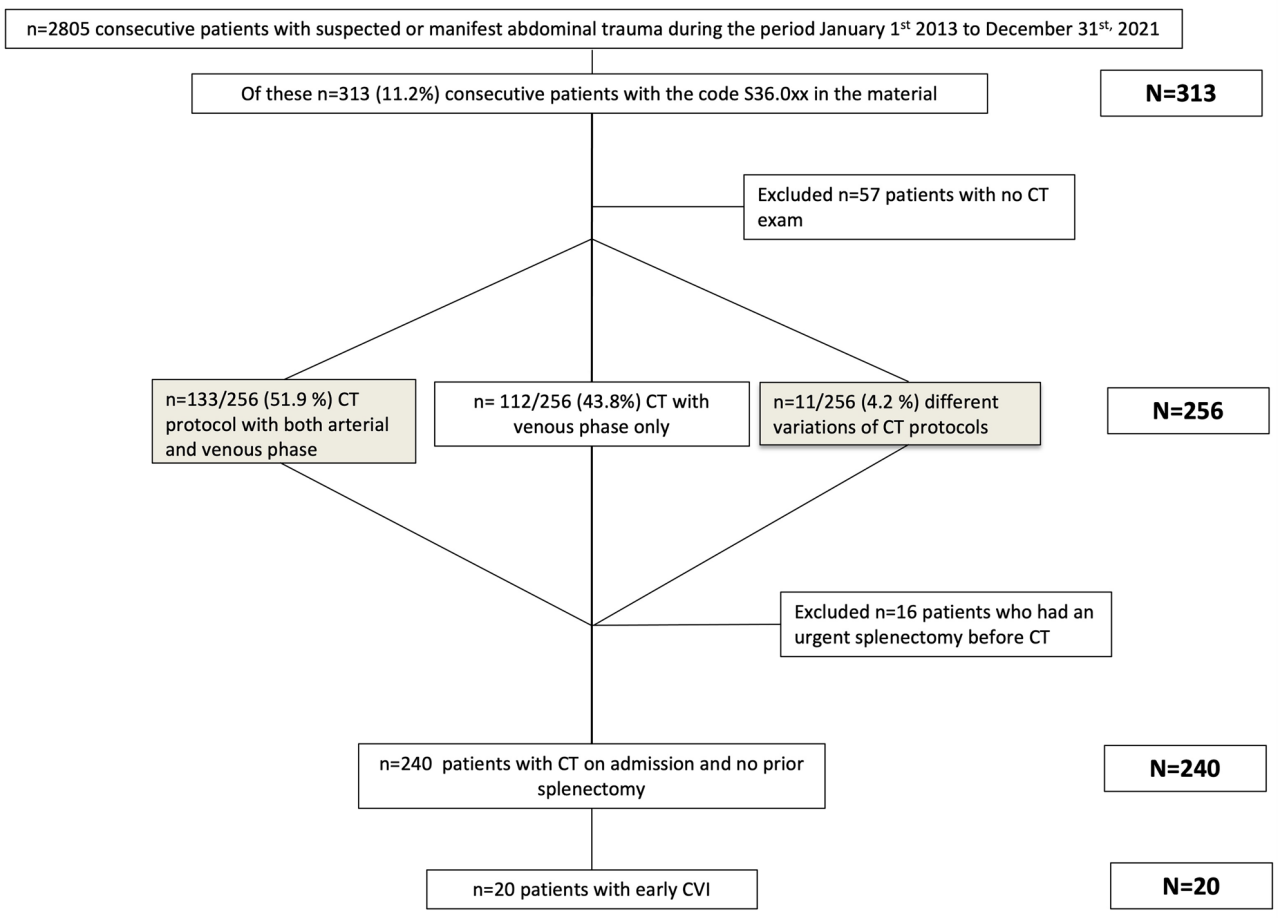
Flowchart of patients with early CVI.

During this study period, splenic CVI was found in 20 patients (Figs. 1, 2). This yields a prevalence of 8.3% (20/240, 95% CI 5.2–12.6%). In addition to information in medical records, a follow-up CT was available in one case, and subsequent conventional angiography in 14 cases. In our material, all splenic CVIs that were identified on angiogram were previously also identified on CT.

In 12 patients (12/20; 60%), only one CVI was seen, two in 4 patients and three in 3 patients. In one patient, multiple small CVIs were seen.

In these patients, the injury mechanism was MVC in 8 cases, fall from height in 6 cases, stabbing injury in 3, undefined blunt trauma to torso, fall on the same level and pedestrian struck by car in 1 case each.

Hospital length of stay was shorter in patients with CVI than in patients without CVI (*p* = 0.0469). No statistically significant differences were found in age, gender, ISS or NISS (Table 8). None of the patients with CVI died within 30 days of the injury.

**Table 8:** Comparison between patients with splenic CVI and no CVI.

	CVI (n = 20)	No CVI (n = 220)	*p*
Gender (M/F)	15/5	169/51	0.8540^a^
Age	43.6 (17–82)	40.1 (15–92)	0.3976^b^
ISS	21 (4–50)	24 (4–66)	0.4936^b^
NISS	25 (8–50)	27 (4–75)	0.2089^b^
LOS days	4.9 (2–20)	12.8 (1–86)	0.0469^b^
Survived to discharge (reciprocal of 30-day mortality)	20 (100%)	207 (94.1%)	

Values are given in mean (age, LOS) or median (ISS, NISS). Range in parentheses.

CVI, contained vascular injury; ISS, Injury Severity Score; NISS, New Injury Severity Score; LOS, hospital length of stay.

^a^Chi-square test.

^b^Mann-Whitney U test.

### CT protocol and CVI

In 18 patients, CVI was detected when both late arterial and venous phase was used. In the two patients, when using a venous phase only, the CVI was confirmed either by conventional angiography or follow-up CT angiography. In cases where both late arterial and venous phase images were available, CVI was seen in 14.5% (18/124, 95% CI 8.6–22.0%).

### Treatment of CVIs

Angioembolization was done in 13 cases (including two out of three cases with a stabbing injury), splenectomy in one. The remaining 6 cases had a non-operative management.

## Discussion

The major aim in this study was to find out the prevalence of early splenic CVI seen on admission CT in a single trauma center’s large unselected material of consecutive patients. In our nine-year series we found a total of 20 patients with splenic CVIs. This yields a prevalence of 8.3%, which is slightly higher than previously reported early splenic CVI prevalence of 3.7–4.7%^18^. It is known that the CT diagnosis of CVI is protocol dependent^13,14^. This is also seen in our study, as 18 out of 20 CVIs were found when both arterial and venous phase was used. As only 51.7% of the CT examinations in this study did include both arterial and venous phase, the prevalence was 14.5% when this protocol was used. This is in accordance with previously reported prevalence of 15%^14,16^. In other words, a substantial number of early CVIs may have been missed because of a suboptimal trauma CT protocol. This might also be the case in the previous study where only venous phase was used^18^. The same may also apply to institutions using the biphasic “split bolus” technique. In one study it was shown that the sensitivity and specificity to diagnose PSA using this technique was 39% and 71%, respectively^15^. However, more studies are needed to verify this as their number of patients (n = 36) was rather small. The change in CT scanner in our hospital most likely did not have impact on the results, since the incidences of both early and latent PSA have previously been shown to remain remarkably stable despite advances in CT technology^18^.

We graded splenic injuries according to AAST OIS 1994 and 2018 systems (Tables 6 and 7). When compared to two other large series^19,20^, the severity spectrum is quite similar when using the 1994 grading system as the most common (40–49%) Grade was III. Also, when using the 2018 grading system, there was a shift towards more Grades IV and V. This reflects the inclusion of CVIs and active bleeding to these higher grades in the 2018 revision.

Delayed splenic CVI, PSA in particular, is not uncommon with a prevalence of 3–20%^18,21–24^. However, if the delayed PSAs really are delayed or simply missed on admission because of a suboptimal CT protocol is still unclear^18,25^. Also, since CVIs are often multiple, 40% in our study, this may increase the risk of delayed bleeding of initially missed CVI further emphasizing the importance of early detection. Rupture of PSA is a rare but well-known complication of blunt splenic trauma with reported incidence of 6%^26,27^. In the majority of cases, it occurs within a week^28,29^, but cases that occur months after trauma have also been reported^30^. In cases of delayed rupture mortality ranges from 5 to 15%^31,32^ which is much higher than the 1% seen in acute splenic injury^33^. On the other hand, it has been shown that regardless of injury grade, early screening leads to a decrease in failure rate emphasizing the importance of early diagnosis^27^. In our study, thirteen patients (65%; 13/20) underwent angioembolization, one underwent splenectomy, and six had a conservative management. The decision on invasive (splenectomy or angioembolization) or conservative treatment was in some cases the result of repeated clinical assessments and other investigations, such as laboratory tests and imaging studies. Conservative management was chosen for those patients who were identified as not to benefit from invasive treatment of the splenic injury.

In a large multi-center study based on radiology reports and medical charts, CVI was reported in 20% of the patients with blunt splenic trauma^16^. However, as the authors point out, their study included selection bias because all participating centers are American College of Surgeons–verified level 1 trauma facilities. As such, findings likely reflect patients seen at similar centers and should not be generalized to all splenic injuries. Also, their data was highly selective, as exclusion criteria included for example penetrating trauma to the abdomen and/or pelvis, CT images obtained more than 12 h before or after initial presentation, splenectomy prior to CT imaging, patients with insufficient follow-up or missing key data, and death before definitive treatment of splenic injury. Similarly, in our study the final study group with CT available and no prior splenectomy included 76.7% of patients with splenic trauma. This may have an impact on the true overall prevalence of CVI, thereby affecting the generalization of our results..

The primary limitation of this study is the retrospective study design from a single trauma center. No additional double-reading was performed, so there is a risk that not all CVIs were identified. However, all CT scans had previously been interpreted by two radiologists and we chose this consensus approach because it mirrors the clinical scenario at our trauma unit where double-reading is the standard of care.

Moreover, not all of the patients had undergone a subsequent angiography that could serve as a reference standard. This also means that we could not differentiate PSA from AV. However, catheter angiography is indicated in either case^34^. In our hospital, all trauma patients with a suspected, or verified, injury to major vessels undergo angiography if it is thought to influence the handling of the patient and if they are clinically fit for the procedure. However, we did not focus on treatment and/or indications for angioembolizations as this is beyond the scope of this retrospective study and treatment protocols vary with time. The use of routine angioembolization for all splenic PSAs has been questioned as there is a high rate of discordance between CT and angiographic identification of splenic PSAs and even when identified at angiogram and embolized, close to half will remain perfused^35^. Also, the authors say that for most isolated splenic PSAs, the natural history of these lesions may be more benign than previously believed. Comparably, the hospital legth of stay was shorter and all patients with CVI in our study survived regardless of the applied treatment.

## Conclusions

In conclusion, the prevalence of early splenic CVI in patients with splenic trauma was 8.3–14.5% (95% CI 5.2–22.0%). Our data suggests that both arterial and venous phase are needed for CT diagnosis. The 30-day outcome in terms of mortality was good.

## Data Availability

The datasets generated and/or analysed during the current study are not publicly available due to sensitive nature of the research and IRB restrictions but are available from the corresponding author on reasonable request.

## Abbreviations

CI: Confidence interval
CVI: Contained vascular injury
PSA: Pseudoaneurysm
AV-fistula: Arterio-venous fistula
ISS: Injury severity score
NISS: New injury severity score
LOS: Length of hospital stay
RIS: Radiological information system
PACS: Picture archiving and communication system
MDCT: Multidetector CT

## References

[1] Bjerke S, Pohlman T, Saywell RM, Przybylski MP, Rodman GH (2006). Evolution, not revolution: Splenic salvage for blunt trauma in a statewide voluntary trauma system—A 10-year experience. Am. J. Surg..

[2] Sladyga A, Benjamin R, Cohn SM (2009). An evidence-based approach to spleen trauma: Management and outcomes. Acute Care Surgery and Trauma: Evidence Based Practice.

[3] Naude G, Bongard FS, Demetriades D (2003). Trauma Secrets: Questions and Answers Reveal the Secrets to Effective Care of the Trauma Patient.

[4] Bessoud B, Denys A, Calmes JM, Madoff D, Qanadli S, Schnyder P (2006). Nonoperative management of traumatic splenic injuries: Is there a role for proximal splenic artery embolization?. Am. J. Roentgenol..

[5] Haan JM, Biffl W, Knudson MM, Davis KA, Oka T, Majercik S (2004). Western Trauma Association Multi-Institutional Trials Committee Splenic embolization revisited: A multicenter review. J. Trauma.

[6] Anderson SW, Varghese JC, Lucey BC, Burke PA, Hirsch EF, Soto JA (2007). Blunt splenic trauma: Delayed-phase CT for differentiation of active hemorrhage from contained vascular injury in patients. Radiology.

[7] Hamilton JD, Kumaravel M, Censullo ML, Cohen AM, Kievlan DS, West OC (2008). Multidetector CT evaluation of active extravasation in blunt abdominal and pelvic trauma patients. RadioGraphics.

[8] Davis, K.A., Fabian. T.C., Croce. M.A., Gavant, M.L., Flick, P.A., Minard, G., *et al*. Improved success in nonoperative management of blunt splenic injuries: embolization of splenic artery pseudoaneurysms. *J. Trauma* **44**, 1008–13; discussion 1013–5 (1998). 10.1097/00005373-199806000-00013 9637156

[9] Di Sabatino A, Carsetti R, Corazza GR (2011). Post-splenectomy and hyposplenic states. Lancet.

[10] Leidner B, Adiels M, Aspelin P, Gullstrand P, Wallén (1998). Standardized CT examination of the multitraumatized patient. Eur. Radiol..

[11] Kozar RA, Crandall M, Shanmuganathan K (2018). AAST Patient Assessment Committee. Organ injury scaling 2018 update: Spleen, liver, and kidney. J. Trauma Acute Care Surg..

[12] Shanmuganathan K, Mirvis SE, Boyd-Kranis R, Zarzaur BL, Coburn M, Cribari C (2000). Nonsurgical management of blunt splenic injury: Use of CT criteria to select patients for splenic arteriography and potential endovascular therapy. Radiology.

[13] Boscak AR, Shanmuganathan K, Mirvis SE, Fleiter TR, Miller LA, Sliker CW (2013). Optimizing trauma multidetector CT protocol for blunt splenic injury: Need for arterial and portal venous phase scans. Radiology.

[14] Uyeda JW, LeBedis CA, Penn DR, Soto JA, Anderson SW (2014). Active hemorrhage and vascular injuries in splenic trauma: Utility of the arterial phase in multidetector CT. Radiology.

[15] Marovic P, Beech PA, Koukounaras J, Kavnoudias H, Goh GS (2017). Accuracy of dual bolus single acquisition computed tomography in the diagnosis and grading of adult traumatic splenic parenchymal and vascular injury. J. Med. Imaging Radiat. Oncol..

[16] Lee JT, Slade E, Uyeda J, Steenbug SD, Chong ST, Tsai R (2021). American Society of Emergency Radiology multicenter blunt splenic trauma study: CT and clinical findings. Radiology.

[17] Brohi K (2008). The Utstein template for uniform reporting of data following major trauma: A valuable tool for establishing a pan-European dataset. Scand. J. Trauma Resusc. Emerg. Med..

[18] Weinberg JA, Lockhart ME, Parmar AD, Griffin RL, Melton SM, Vandromme MJ (2010). Computed tomography identification of latent pseudoaneurysm after blunt splenic injury: Pathology or technology?. J. Trauma.

[19] Margari S, Garozzo Velloni F, Tonolini M, Colombo E, Artioli D, Allievi NE (2018). Emergency CT for assessment and management of blunt traumatic splenic injuries at a Level 1 Trauma Center: 13-year study. Emerg. Radiol..

[20] Morell-Hofert D, Primavesi F, Fodor M, Gassner E, Kranebitter V, Braunwarth E (2020). Validation of the revised 2018 AAST-OIS classification and the CT severity index for prediction of operative management and survival in patients with blunt spleen and liver injuries. Eur. Radiol..

[21] Weinberg, J.A., Magnotti, L.J., Croce, M.A., Edwards, N.M., Fabian, T.C. The utility of serial computed tomography imaging of blunt splenic injury: Still worth a second look? *J. Trauma* **62**, 1143–7; discussion 1147–8 (2007). 10.1097/TA.0b013e318047b7c2 17495714

[22] Wallen TE, Clark K, Baucom MR, Pabst R, Lemmink J, Pritts TA (2020). Delayed splenic pseudoaneurysm identification with surveillance imaging. J. Trauma Acute Care Surg..

[23] Muroya T, Ogura H, Shimizu K, Tasaki O, Kuwagata Y, Fuse T (2013). Delayed formation of splenic pseudoaneurysm following nonoperative management in blunt splenic injury: Multi-institutional study in Osaka, Japan. J. Trauma Acute Care Surg..

[24] Poletti PA, Becker CD, Arditi D, Terraz S, Buchs N, Shanmuganathan K (2013). Blunt splenic trauma: Can contrast enhanced sonography be used for the screening of delayed pseudoaneurysms?. Eur. J. Radiol..

[25] Davis, K.A., Fabian, T.C., Croce, M.A., Gavant, M.L., Flick, P.A., Minard, G., *et al*. Improved success in nonoperative management of blunt splenic injuries: embolization of splenic artery pseudoaneurysms. *J. Trauma* **44**, 1008-13; discussion 1013-5 (1998). 10.1097/00005373-199806000-00013 9637156

[26] Sabe AA, Claridge JA, Rosenblum DI, Lie K, Malangoni MA (2009). The effects of splenic artery embolization on nonoperative management of blunt splenic injury: A 16-year experience. J. Trauma.

[27] Leeper WR, Leeper TJ, Ouellette D, Moffat B, Sivakumaran T, Charyk-Stewart T, Kribs S, Parry NG, Gray DK (2014). Delayed hemorrhagic complications in the nonoperative management of blunt splenic trauma: Early screening leads to a decrease in failure rate. J. Trauma Acute Care Surg..

[28] Gavant ML, Schurr M, Flick PA, Croce MA, Fabian TC, Gold RE (1997). Predicting clinical outcome of nonsurgical management of blunt splenic injury: using CT to reveal abnormalities of splenic vasculature. Am. J. Roentgenol..

[29] Santorelli JE, Costantini TW, Berndtson AE, Kobayashi L, Doucet JJ, Godat LN (2022). Readmission after splenic salvage: How real is the risk?. Surgery.

[30] Maria C, Nickolaos T, George G, Michail K (2021). Delayed splenic rupture 4 months following minor blunt abdominal trauma. Glob. J. Rare Dis..

[31] Kluger Y, Paul DB, Raves JJ, Fonda M, Young JC, Townsend RN (1994). Delayed rupture of the spleen—Myths, facts, and their importance: Case reports and literature review. J. Trauma.

[32] Foster RP (1970). Delayed haemorrhage from the ruptured spleen. Br. J. Surg..

[33] Cogbill TH, Moore EE, Jurkovich GJ, Morris JA, Mucha P, Shackford SR (1989). Nonoperative management of blunt splenic trauma: A multicenter experience. J. Trauma.

[34] Fleiter TR, Archer-Arroyo K, Mirvis SE, Soto JA, Shanmuganathan K, Yu J, Kubal WS (2014). Blunt abdominal and retroperitoneal trauma. Problem Solving in Emergency Radiology E-Book.

[35] Radding S, Harfouche MN, Dhillon NK, Ko A, Hawley KL, Kundi R (2023). A pseudo-dilemma: Are we over-diagnosing and over-treating traumatic splenic intraparenchymal pseudoaneurysms?. J. Trauma Acute Care Surg..

